# Marine Purple Photosynthetic Bacteria as Sustainable Microbial Production Hosts

**DOI:** 10.3389/fbioe.2019.00258

**Published:** 2019-10-11

**Authors:** Mieko Higuchi-Takeuchi, Keiji Numata

**Affiliations:** Biomacromolecules Research Team, RIKEN Center for Sustainable Resource Science, Saitama, Japan

**Keywords:** purple photosynthetic bacteria, polyhydroxyalkanoate, extracellular nucleic acids, hydrogen, sustainable production

## Abstract

Photosynthetic microorganisms can serve as the ideal hosts for the sustainable production of high-value compounds. Purple photosynthetic bacteria are typical anoxygenic photosynthetic microorganisms and are expected to be one of the suitable microorganisms for industrial production. Purple photosynthetic bacteria are reported to produce polyhydroxyalkanoate (PHA), extracellular nucleic acids and hydrogen gas. We characterized PHA production as a model compound in purple photosynthetic bacteria, especially focused on marine strains. PHA is a family of biopolyesters synthesized by a variety of microorganisms as carbon and energy storage materials. PHA have recently attracted attention as an alternative to conventional petroleum-based plastics. Production of extracellular nucleic acids have been studied in *Rhodovulum sulfidophilum*, a marine purple non-sulfur bacterium. Several types of artificial RNAs have been successfully produced in *R. sulfidophilum*. Purple photosynthetic bacteria produce hydrogen via nitrogenase, and genetic engineering strategies have been investigated to enhance the hydrogen production. This mini review describes the microbial production of these high-value compounds using purple photosynthetic bacteria as the host microorganism.

## Introduction

Biosynthesis of high-value compounds in photosynthetic organisms is one of the potential methods to reduce costs, and can contribute to a sustainable system because they can utilize sunlight energy and carbon dioxide (CO_2_) in the air for their growth. Cyanobacteria, algae and plants have two photosystems (photosystem I and II), extract electrons from water, and evolve oxygen as a byproduct (Fischer et al., 2016). On the other hand, anoxygenic photosynthetic bacteria possess only a single photosystem, either type I or type II photosynthetic reaction center, and extract electrons from organic compounds, sulfur compounds and hydrogen. Since anoxygenic type I and type II reaction centers are structurally and functionally similar to oxygenic photosystems, a lot of progress in photochemical reaction, and electron transport in photosynthetic reaction centers, has been made using anoxygenic photosynthetic bacteria owing to their simple structure (Hillier and Babcock, 2001).

Purple photosynthetic bacteria, which are typical anoxygenic photosynthetic bacteria, are classified into purple sulfur and purple non-sulfur bacteria. Purple sulfur bacteria use sulfide and hydrogen as an electron donor, whereas purple non-sulfur bacteria utilize organic compounds (Madigan and Jun, 2009). Purple photosynthetic bacteria are widely distributed in aquatic environments. We succeeded the isolation of marine purple non-sulfur bacteria from natural seawater (Higuchi-Takeuchi et al., 2016). Some species of purple non-sulfur bacteria are known to have the nitrogen fixation ability (McKinlay and Harwood, 2010). This means that they can use N_2_ in the air as nitrogen source for their growth. However, exact nitrogen fixation ability of purple non-sulfur bacteria has not been evaluated despite their contribution to the nitrogen flux in aquatic environments.

**Figure 1:**
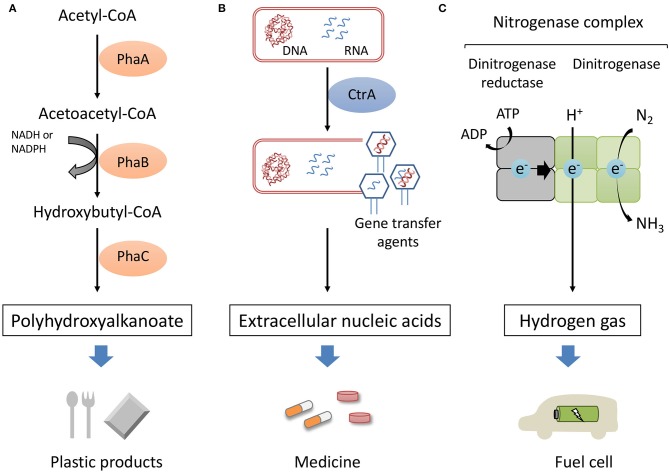
Sustainable production of high-value compounds in marine purple photosynthetic bacteria. PHA was produced from acetyl-CoA in tree steps **(A)**. Extracellular nucleic acids were produced in the process of GTA-like particle production controlled by CtrA **(B)**. The nitrogenase complex is composed of dinitrogenase reductase and dinitrogenase. Nitrogenase catalyzes the proton reduction to hydrogen as well as the reduction of nitrogen to ammonia **(C)**.

The utilization of marine organisms has several potential advantages for large-scale commercial production. Sterilized seawater can be used as a culture medium instead of a synthetic medium. Moreover, the high salt concentration of seawater can inhibit biological contamination during the cultivation. Considering these advantages, marine purple photosynthetic bacteria would be an ideal host organism for microbial production. Purple photosynthetic bacteria are reported to produce intracellular polyhydroxyalkanoate (PHA), extracellular nucleic acids and hydrogen gas as shown in Figure 1. In this mini review, we summarize the current state of biological production using marine purple photosynthetic bacteria as host microorganisms.

## PHA Production

PHA is a family of biopolyesters that a lot of microorganisms accumulate as carbon and energy storage materials in the presence of excess carbon (Lenz and Marchessault, 2005). PHA have attracted attention due to their biodegradable and biocompatible properties (Numata et al., 2009). *Cupriavidus necator*, a hydrogen-oxidizing bacterium, is the most studied bacterium for PHA production and produced about 90% of dry cell weight (wt%) PHA (Steinbuchel, 1991).

One of the most significant factors for commercial PHA production is the cost for carbon sources such as sugars or plant oils. In addition, the supply of those carbon sources is not stable because of natural environmental factors such unexpected weather and natural disasters. To solve the problems, direct production of PHA from CO_2_ via photosynthesis has been investigated using cyanobacteria and plants (Osanai et al., 2013; Yoshizumi et al., 2017). Among photosynthetic organisms, purple photosynthetic bacteria are known to have better ability to produce PHA (Liebergesell et al., 1991). PHA production in purple photosynthetic bacteria has been characterized using freshwater-type purple photosynthetic bacteria such as *Rhodospirillum rubrum* (Brandl et al., 1989), *Rhodobacter sphaeroides* (Khatipov et al., 1998), *Rhodobacter capsulatus* (Kranz et al., 1997), and *Allochromatium vinosum* (Rehm and Steinbuchel, 1999). On the other hand, reports about PHA production using marine purple bacteria are limited, even though marine bacteria have several advantages, as described in this review. Therefore, we evaluated for the production of PHA by marine purple photosynthetic bacteria and found that 3 purple sulfur bacteria and 9 purple non-sulfur bacteria strains synthesized PHA (Higuchi-Takeuchi et al., 2016).

### PHA Synthase of Purple Photosynthetic Bacteria

PHA is produced from acetyl-coenzyme A (CoA) through three enzyme reactions (Figure 1A). Ketothiolase (PhaA) catalyzes the formation of acetoacetyl-CoA from two acetyl-CoA molecules. Acetoacetyl reductase (PhaB) reduce acetoacetyl-CoA to 3-hydroxyacyl-CoA. PHA synthase (PhaC) catalyzes the polymerization of (R)-3-hydroxyacyl-CoA to PHA. PhaC is a key enzyme of PHA synthesis and polymerization reaction of PhaC have been studied extensively (Stubbe and Tian, 2003; Numata et al., 2012, 2015). Crystal structures of the catalytic domain of PhaC from *R. eutropha* and *Chromobacterium* sp. USM2 were reported (Kim et al., 2016; Wittenborn et al., 2016; Chek et al., 2017). However, detailed PHA polymerization mechanism is still not completely elucidated.

PHA synthase is divided into four classes according to subunit composition, sequence similarity and substrate specificity, as shown in Table 1 (Rehm, 2003; Stubbe and Tian, 2003; Stubbe et al., 2005). Classes I and II consist of single subunit PhaC with molecular weight between 60 and 70 kDa. Class III synthases are composed of two subunits, PhaC and PhaE. Class IV synthases are composed of the PhaC of PhaR subunits. We checked PhaC amino acid sequences from 21 purple photosynthetic bacteria strains that whole genome sequences are available. PhaC from 13 purple non-sulfur bacteria were categorized as Class I PHA synthase. On the other hand, PhaC and PhaE homologous sequences were found from 8 purple sulfur bacteria strains, indicating that PHA synthase belong to Class III. *A. vinosum*, a purple sulfur bacterium, has Class III type PhaC and extensively studied its biological activity (Liebergesell and Steinbuchel, 1992; Rehm and Steinbuchel, 1999; Yuan et al., 2001). *Rhodovulum sulfidophilum* is a marine purple non-sulfur bacterium and widely used as a representative strain. Whole genome sequences of *R. sulfidophilum* were determined in 2013 (Masuda et al., 2013). We discovered *phaC* homologous sequences in the *R. sulfidophilum* was classified as a class I PhaC. Alignment analysis of amino acids revealed that important amino acid residues for PHA polymerization were conserved in *R. sulfidophilum*.

**Table 1:** Classification of PHA synthase.

**Class**	**Subunits (MW)**	**Substrate**	**Organism (References)**
I	PhaC (60–73 kDa)	C3–C5 monomer	*Cupriavidus necator* (Yuan et al., 2001)*, Aeromonas caviae* (Numata et al., 2012)
II	PhaC (60–65 kDa)	>C6 monomer	*Pseudomonas* sp. 61-3 (Takase et al., 2004)
III	PhaC (40–53 kDa) PhaE (20–40 kDa)	C3–C5 monomer	*Allochromatium vinosum* (Muh et al., 1999)*, Synechocystis* sp. PCC6803 (Numata et al., 2015)
IV	PhaC (40 kDa) PhaR (22 kDa)	C3–C5 monomer	*Bacillus megaterium* (McCool and Cannon, 2001)

PHA synthase from *R. sulfidophilum* (PhaC_Rs_) was produced by a cell free protein expression system and characterized its activity (Higuchi-Takeuchi et al., 2017). The polymerization activity of PhaC_Rs_ increased linearly with increasing concentrations of substrate, (*R*)-3-hydroxybutyryl-CoA (3HB-CoA) and did not saturate, suggesting that the PhaC_Rs_ was not saturated due to low affinity for the substrate. Generally, PhaC is thought to exist as monomeric and dimeric forms in equilibrium and dimerization of PhaC induced by substrate binding facilitate the PHA polymerization (Wodzinska et al., 1996). We analyzed multimer formation of PhaC_Rs_ by size exclusion chromatography and Native PAGE, the results of which showed PhaC_Rs_ existed predominantly as a dimer form even in the absence of 3HB-CoA (Higuchi-Takeuchi et al., 2017). Dimerization of PhaC is considered to be the rate-limiting steps for PHA polymerization. Linear relationship between the PhaC_Rs_ activity and concentrations of 3HB-CoA might result from low affinity for the substrate and the absence of rate-limiting step due to the existence of predominant active dimer. These properties are quite different from well-known PhaC.

### PHA Production Under Various Culture Conditions

PHA accumulation is known to be enhanced in the presence of excess carbon and under nutrient limited conditions such as nitrogen and phosphorus (Lenz and Marchessault, 2005). Nitrogen limited conditions were commonly used for PHA production in the case of purple photosynthetic bacteria. We examined PHA production under nutrient rich and nitrogen limited conditions and found a difference between purple sulfur bacteria and purple non-sulfur bacteria (Higuchi-Takeuchi et al., 2016). Marine purple sulfur bacteria synthesized PHA only under nitrogen-limited conditions and the yield of PHA was 50–200 mg/L. In contrast, marine purple non-sulfur bacteria were able to produce PHA under growth conditions without nutrient deficiency. Under this condition, one marine purple non-sulfur bacteria produced 302 mg PHA /L. PHA-producing bacteria are classified into two groups according to culture nutrient conditions (Lee, 1996). The first group bacteria require nutrient limitation for PHA production. A lot of PHA-producing bacteria including *C. necator* belong to this group. In the second group bacteria, nutrient limitation is not required for PHA production. Marine purple sulfur bacteria are categorized to the first group, whereas purple non-sulfur bacteria belong to the second group. We also found that iron concentrations (ferric citrate) affect the cell growth and PHA production in *R. sulfidophilum* (Foong et al., 2019). Very low concentrations of iron (1–2 μM) was sufficient to promote cell growth and a high PHA yield (1,000 mg/L) during the logarithmic phase.

PHA production was examined in marine purple non-sulfur bacteria under various growth light and oxygen conditions (Higuchi-Takeuchi and Numata, 2019). *R. sulfidophilum* produced higher PHA under low-light conditions than under high-light conditions. The 800-nm LED lighting was the best for PHA concentration (1,200 mg/L) among three types of wavelengths we studied. We found that marine purple non-sulfur bacteria strains hardly accumulated PHA (<5 wt%) under aerobic conditions in the presence of malate and pyruvate. Interestingly, the addition of acetate induced high PHA production (33 wt%) under aerobic conditions. The expression of isocitrate dehydrogenase in the tricarboxylic acid (TCA) cycle decreased under aerobic conditions in the presence of malate and pyruvate and upregulated by the addition of acetate. Considering these results, we proposed that low PHA production under aerobic conditions is caused by low activity of the TCA cycle and its activity was enhanced by the addition of acetate. We found that the expression of PdhR, which is a transcriptional repressor of the pyruvate dehydrogenase complex, was upregulated upon the addition of acetate. The changes in the metabolic state might be induced by the addition of acetate under aerobic conditions and PdhR is involved in this regulation.

### PHA Properties Synthesized in Purple Photosynthetic Bacteria

Microorganisms can produce various type of PHAs depending on the carbon source and metabolic pathway and more than 150 monomer unit has been identified to date. The most common types of monomer are 3-hydroxybutyrate (3HB) and 3-hydroxyvalerate (3HV). PHA composition affects the mechanical and thermal properties of PHA. Poly(3-hydroxybutyrate) [P(3HB)], homopolymer of 3HB, is a highly crystalline and brittle material. Melting temperature of P(3HB) is around 180°C (Rehm, 2003). The copolymer of 3HB and 3HV, Poly(3-hydroxybutyrate-*co*-3-hydroxyvalerate) [P(3HB-*co*-3HV)], has a lower melting temperature and higher biodegradability compared to P(3HB) depending on the polymer composition (Mitomo et al., 1995). We found that three strains of marine purple sulfur bacteria synthesized 3HB homopolymer (Higuchi-Takeuchi et al., 2016). On the other hand, purple non-sulfur bacteria synthesized copolymers of 3HB and 3HV. The similar copolymer syntheses were reported in freshwater type purple photosynthetic bacteria (Liebergesell et al., 1991). PHA synthases of purple sulfur bacteria (Class III) and purple non-sulfur bacteria (Class I) are different as described above. Differences of PHA production and properties might be explained by different properties of PHA synthase.

Molecular weight and its distributions are important properties of polymer production, because it affects physical and mechanical characteristics of polymeric materials. In the case of PHA, decreases in molecular weight of PHA have been reported during extraction and purification processes (Ramsay et al., 1990; Hahn et al., 1994). In addition, higher molecular weights PHA are known to have desirable mechanical properties (Aoyagi et al., 2003). Therefore, high-molecular weight PHA production has been studied using *E. coli* that do not have PHA degradation pathway (Kusaka et al., 1999). Gel permeation chromatography analysis revealed that some marine purple photosynthetic bacteria strains synthesized high-molecular-weight PHA compared to other PHA-producing bacteria (Higuchi-Takeuchi et al., 2016). Thus, PHA produced by purple photosynthetic bacteria has valuable properties for industrial PHA production.

## Extracellular Nucleic Acid Production

Extracellular nucleic acids (DNA and RNA) have been found in natural conditions such as freshwater, seawater, and soil and it is reported that some bacteria produced nucleic acids extracellularly (Paul and David, 1989; Vlassov et al., 2007). These extracellular nucleic acids are proposed to have a role in biofilm formation and horizontal gene transfer that is the movement of genetic information between organisms. *R. sulfidophilum* is one of the bacteria that produce extracellular nucleic acids (Ando et al., 2006; Suzuki et al., 2009). One group extensively studied extracellular nucleic acids in *R. sulfidophilum* (Kikuchi and Umekage, 2018). They found that log phase cells of *R. sulfidophilum* produced extracellular nucleic acids in the culture media (Ando et al., 2006). Extracellular DNA sequences were found in their genome (Suzuki et al., 2009) and extracellular soluble RNAs corresponded to the ribosomal RNAs and transfer RNAs (Ando et al., 2006).

Gene transfer agents (GTAs) are considered to be involved in the production of extracellular nucleic acids in *R. sulfidophilum*. GTAs are bacteriophage-like particles that package DNA fragments and were first discovered in *R. capsulatus* (Lang et al., 2012). The genes with homology to the GTA components were identified in the genome of *R. sulfidophilum* and GTA-like particles were found in *R. sulfidophilum* cell cultures (Nagao et al., 2015). The two-component signal transduction protein, CtrA, has been reported to be necessary for the GTAs of *R. capsulatus* (Lang and Beatty, 2000). The *ctrA*-deficient mutant of *R. sulfidophilum* lost the ability to produce GTA-like particles and also decreased the amount of extracellular soluble nucleic acids (Komatsu et al., 2018). Thus, extracellular nucleic acid production is involved in GTA-like particle production and controlled by CtrA (Figure 1B).

### Production of Artificial RNA in *R. sulfidophilum*

Variety types of RNA molecules have been reported to date, such as small interfering RNAs, double-stranded RNAs, piwi-interacting RNAs and micro RNAs. RNAs have become key players in biology and have also utilized as medicines for RNA-based therapy. Currently, these RNA molecules have been prepared by *in vitro* transcription (Milligan et al., 1987) and chemical synthesis (Marshall and Kaiser, 2004). However, these methods are expensive and time-consuming and not appropriate for large quantities of RNAs. Alternative method for RNA production is a microbial production.

*R. sulfidophilum* is a good microbial host for RNA production because this bacterium has no detectable ribonucleases (Suzuki et al., 2010). Using *R. sulfidophilum, in vivo* production methods of artificial RNA were reported (Suzuki et al., 2010, 2011; Nagao et al., 2014). They succeeded the production of 45 ng/L streptavidin RNA aptamers that function as an RNA drug by specifically targeting a defined molecule (Suzuki et al., 2010). The RNA aptamers were produced in the culture medium and retained streptavidin binding ability. Production of RNA aptamers was improved by modification of promoter. Finally, the extracellular RNA aptamer of 200 ng could be prepared from 1 L culture (Suzuki et al., 2011). They also produced the short hairpin RNA that contain long stem-loop structure (Nagao et al., 2014). The other group succeeded production of human microRNA precursor in *R. sulfidophilum* (Pereira et al., 2016).

## Photohydrogen Production

Hydrogen gas is a completely clean-burning fuel. However, most of the hydrogen is produced from fossil fuels (Holladay et al., 2009). Photosynthetic organisms convert H_2_O, reduced sulfur compounds and organic compounds into hydrogen utilizing sunlight energy (photohydrogen production). Purple non-sulfur bacteria is known to produce hydrogen via nitrogenase (McKinlay and Harwood, 2010; Eroglu and Melis, 2011) (Figure 1C). Nitrogenase are composed of two multisubunit proteins, dinitrogenase reductase and dinitrogenase. Dinitrogenase reductase transfers electrons to the dinitrogenase with concomitant ATP hydrolysis. Nitrogenase mediates the reduction of nitrogen gas into ammonia and protons into molecular hydrogen. Hydrogen is produced as a by-product of the nitrogenase reaction according to Equation (1):

(1) $$\begin{array}{l} {\text{N}}_{2}+8{\text{H}}^{+}+8{\text{e}}^{-}+16\text{ATP}\to 2\text{N}{\text{H}}_{3}+{\text{H}}_{2}+16\text{ADP}+16\text{Pi} \end{array}$$

Nitrogenase complex is known to be very sensitive to oxygen. Since purple non-sulfur bacteria extract electrons from substrates other than water such as organic carbon, oxygen is not produced during the photosynthesis. In addition, purple non-sulfur bacteria can grow under anaerobic conditions. Therefore, nitrogenase complex is not inhibited by oxygen in anaerobic grown purple non-sulfur bacteria. *R. palustris* (Rey et al., 2007) and *R. sphaeroides* (Koku et al., 2002) are studied as well as host strains of purple non-sulfur bacteria for photohydrogen production. *R. sulfidophilum* is also reported to have hydrogen production ability (Maeda et al., 2003; Cai and Wang, 2012).

### Improvement of Photohydrogen Production in Purple Non-sulfur Bacteria

Genetic manipulation has been applied to improve photohydrogen production of purple non-sulfur bacteria. Hydrogenase catalyze the oxidization of hydrogen to reuse hydrogen, leading to consumption of hydrogen. Therefore, one target of enhanced hydrogen production is inactivation of hydrogenase. For example, hydrogenase-knockout mutant of *R. sphaeroides* produced 2.42 L H_2_/L culture (Kars et al., 2009) and *R. capsulatus* produced 0.14 mL H_2_/h/mg dry cell weight (Ooshima et al., 1998). Photosynthetic organisms have a light harvesting system consisting of proteins and pigments to absorb light. The light harvesting system changes their size to absorb light efficiently depending on light environments. The light harvesting size was reduced to increase light capture efficiency and this mutant showed 1.4 folds higher hydrogen production (Kondo et al., 2002). Another target for the enhancement of hydrogen production is the modification of PHA synthesis. It is considered that hydrogen production competes with PHA synthesis in terms of reducing power. PHA synthesis deletion mutant of *R. sphaeroides* showed higher hydrogen production (3.34 mL H_2_/mg dry cell weight) (Hustede et al., 1993; Kim et al., 2006).

A variety of kinds of large-scale photobioreactor has been investigated for industrial photohydrogen production in purple non-sulfur bacteria. Reactor design, culture light condition and nutrient sources have been investigated and are reviewed elsewhere (Eroglu and Melis, 2011). Since purple non-sulfur bacteria can utilize waste materials containing organic carbon as carbons source, photohydrogen production was investigated using various kind of wastes to reduce production cost (Wu et al., 2012). Hydrogen production could be successfully achieved using waste water from manufacturer and kitchen (118 mL H_2_/h) (Tao et al., 2008) and food wastes (2.75 mL H_2_/g dry cell weight, 40 L H_2_/L) (Franchi et al., 2004; Laurinavichene et al., 2010).

## Conclusion and Perspectives

Marine purple photosynthetic bacteria are environmentally friendly microorganisms and can produce high-valuable compounds such as PHA, extracellular nucleic acids, and hydrogen gas. Previously, we demonstrated that marine purple photosynthetic bacteria were able to produce PHA even in seawater (Higuchi-Takeuchi et al., 2016), suggesting that abundant natural resources such as seawater, CO_2_, N_2_ and sunlight energy can be used as a culture medium, biological sources and energies. Genetic tools such as synthetic promoter and transformation method have not been fully established yet in marine purple photosynthetic bacteria, even though we recently developed a transformation method using chemical competent cells of marine purple photosynthetic bacteria (Higuchi-Takeuchi et al., 2019). In addition, large-scale, continuous, and high cell density cultivation methods for ideal photosynthetic production have not been established yet. However, we are interested in multiple advantages of marine purple photosynthetic bacteria over the other microbial systems as described in this mini-review and also seriously consider that marine purple photosynthetic bacteria would be a suitable production host contributing to the sustainable society in future.

## Author Contributions

MH-T and KN conceptualized and wrote the manuscript.

### Conflict of Interest

The authors declare that the research was conducted in the absence of any commercial or financial relationships that could be construed as a potential conflict of interest.
